# Disseminating skills to carers of people with eating disorders: an examination of treatment fidelity in lay and professional carer coaches

**DOI:** 10.1080/21642850.2014.908716

**Published:** 2014-04-28

**Authors:** Pamela Macdonald, Rebecca Hibbs, Charlotte Rhind, Amy Harrison, Elizabeth Goddard, Simone Raenker, Gill Todd, Janet Treasure

**Affiliations:** ^a^ Eating Disorders Unit, Institute of Psychiatry, King's College London, The Basement, PO59, 103 Denmark Hill, LondonSE5 8AF, UK; ^b^ South London & Maudsley NHS Trust, London, UK

**Keywords:** eating disorders, anorexia nervosa, carer support, skills training programmes, carer coaching

## Abstract

Family members of people with eating disorders (EDs) have high levels of stress and can use maladaptive methods of coping. We have developed an intervention, using motivational interviewing (MI) strategies that trains lay and professional carer coaches (CCs) to support carers of adolescents with EDs to use more adaptive coping procedures. The aim of this study is to measure treatment integrity in coaches with either academic or lived experience. Eleven coaches were trained and supervised by an expert trainer and an ‘expert by experience’ trainer. Six of the coaches had prior training in clinical work and/or psychology and five had personal experience of supporting a loved one with an ED. Two audio-taped sessions (Sessions 3 and 7) from each family coached (*n* = 22) were assessed for fidelity to MI. Half the sessions (50% *n* = 11) had a Motivational Interviewing Treatment Integrity global score above the suggested cut-off for recommended competency. Prior clinical training was related to higher treatment fidelity and experiential training (having coached a greater number of families) improved treatment fidelity in the lay carer group. These preliminary findings suggest that: “lay CCs” can be trained to deliver an intervention based on MI. Further exploration of a more effective means of training, monitoring and supervision is required to maximise the quality of the intervention.

## 1. Background

The NICE guidelines in the UK recommend that most people with eating disorders (EDs) be managed on an outpatient basis (National Institute for Clinical Excellence, 2004), which frequently results in family members providing a large amount of care (Padierna et al., 2013; Raenker et al., 2013). The consequences for carers in terms of burden, anxiety and depression have been summarised in a systematic review (Zabala, Macdonald, & Treasure, 2009) and meta-analyses (Anastasiadou, Medina-Pradas, Sepulveda, & Treasure, 2014). Commonly reported feelings include guilt (Goldner & Birmingham, 1994), frustration (Kamerling & Smith, 2010) and not being able to cope (Perkins, Winn, Murray, Murphy, & Schmidt, 2004). Maladaptive coping strategies are thought to contribute to the stress of caregiving (Coomber & King, 2012). However, these appraisals can be improved by providing carers with information and skills sharing. A recent systematic review and meta-analyses found that carer distress and burden can be reduced by a variety of psychoeducational interventions and delivered at a minimal level of professional resource (Hibbs, Rhind, Leppanen, & Treasure, 2014), including two interventions that taught specific skills to produce behaviour change such as motivational interviewing (MI) (Goddard et al., 2011; Hibbs et al., 2014).

Carers themselves can be ambivalent about change and often report lacking the skills and resources required to care for their loved ones (Haigh & Treasure, 2003; Treasure et al., 2001). Our team has developed an intervention, Experienced Carers Helping Others (ECHO), that can deliver training to carers to enhance their knowledge and skills, decrease their level of distress and improve coping, as well as provide them with limited, knowledgeable support in an accessible and interactive way. ECHO consists of a book (Treasure, Smith, & Crane, 2007), 5 DVDs (3 theoretical and 2 practical) and 10 telephone coaching sessions that are allocated, where possible, between participating carers (e.g. mother and father). The aim of telephonic coaching is to provide a supplementary interpersonal element to the materials by helping carers evaluate their current situation, encourage motivation and commitment and look at any area where they may be able to experiment with the skills, perhaps by setting an action plan. It also helps to model communication skills that both address symptom management and help families change attitudes and behaviours that serve to maintain the disorder (Treasure, Lopez, & Macdonald, 2011). Coaches are trained in the maintenance model of carer stress/coping and expressed emotion (Schmidt & Treasure, 2006), later modified to include accommodating behaviours and maladaptive coping as maintaining factors (Treasure & Schmidt, 2013), as a template for targeting problematic behaviours and possible areas for change. The maintenance model proposes that interpersonal factors such as interactions, communication patterns and accommodating and enabling behaviours can have an impact on the course of the ED. MI is the primary therapeutic tool used to deliver the telephone coaching (Rollnick, Butler, Kinnersley, Gregory, & Mash, 2010) and is a collaborative, goal-oriented style of communication designed to strengthen personal motivation for commitment to a specific goal by eliciting and exploring the person's own reasons for change within an atmosphere of acceptance and compassion (Miller & Rollnick, 2013). Coaches are trained to promote change through the recognition of ambivalence in the individual, reflective listening and eliciting change talk; encouraging carers to identify, reflect on and change their own behaviours. The primary role of the coach is to support the carer in a warm, non-judgemental and empathetic approach by using the principles of MI. The telephone coaches are volunteers with personal or professional experience of EDs. We trained carer coaches (CCs) from two sources: (1) those with the lived experience of EDs, based on the ethos of the expert–patient and recovery models (Department of Health, 2001; The Royal College of Psychiatrists and Social Care Institute for Excellence: Joint Position Paper, 2007) and (2) individuals professionally affiliated to the field of EDs.

There is preliminary support for a treatment effect of ECHO on individuals with EDs. The self-management materials were supplemented with three coaching sessions and carers reported a functional improvement in the people they were caring for and this was associated with reductions in expressed emotion and accommodating and enabling behaviours (Goddard et al., 2011). Moreover, in ED treatment generally, the addition of guidance improves the effectiveness of self-help (Grover et al., 2011a, 2011b) and there are promising results in other health-related fields for trained peer support delivered by telephone (Dale, Caramlau, Docherty, Sturt, & Hearnshaw, 2007; Ghorob et al., 2011; Goldman, Ghorob, Eyre, & Bodenheimer, 2013; McCusker et al., 2012). However, as yet, neither professionals nor carers' skills have been measured in terms of fidelity to the intervention or guidance being offered.

Miller and Rollnick (2014) raise legitimate concerns as to whether a treatment or intervention contains an adequate dose of what it purports to deliver, whether the providers are trained to an appropriate level of competence and whether the intervention is being delivered with fidelity sufficient to evaluate an effect. Therefore, in order to examine whether a peer-support model is feasible, it is important to establish whether lay coaches with minimal levels of psychological training could reach an acceptable level of proficiency with training and supervisory procedures in place. Consequently, the aim of this study is to examine whether coaches with varied affiliations to EDs (those with lived and/or professional expertise in EDs) can be trained to an acceptable level of MI proficiency.

## 2. Method

Examination of treatment fidelity in the present study was completed within the context of a multi-centre randomised controlled trial (RCT; ISRCTN83003225) evaluating a skill-based intervention for carers of someone with anorexia nervosa (AN) (Experienced Carers Helping Others [ECHO]) (Rhind et al., 2014). Carers (*n* = 49) of adolescents with a primary diagnosis of AN according to Diagnostic and Statistical Manual of Mental Disorders, Fourth Edition (DSM-IV) criteria (American Psychiatric Association, 1994) or EDs not otherwise specified AN type (EDNOS-AN) receiving outpatient treatment were offered 10 telephone coaching sessions that were allocated, where possible, between participating carers (e.g. mother and father). Coaches were asked to complete the sessions within a five-month period, ideally carrying out telephone sessions at two weekly intervals. Ethical approval was granted by the Northwick Park Hospital Ethics Committee (CREC ref no. 11/H0724/4).

### 2.1. Coaches

Inclusion criteria were experience of caring for people with AN (family or professional), having previously participated in a skill-based carer intervention, having sufficient time for the training, supervision and coaching and access to computers for the distance learning element as well as for email supervision and communication purposes. Exclusion criteria were having a family member with AN who remains acutely ill, current diagnosis of an ED themselves and English non-fluency. We trained 11 coaches from 2 sources: (1) lay CCs with the lived experience of EDs and (2) those with a professional affiliation to the field of EDs. Table 1 provides a detailed account of the coaches' backgrounds.

**Table 1.** Demographics.

ID number	Previous experience in delivering therapy	Recovery stage of own child/self	Duration of illness	Number of years in recovery	Currently living with sufferer	Number of families coached
CCs						
CC1	No	Maintenance	8 years	7	Yes	14
CC2	No	Maintenance/recovered	10 years	5	No	8
CC3	No	Maintenance	20+ years	5	Yes	6
CC4	Running support group	Recovered	2 years	10	No	7
CC5	Running support group	Fatality	5 years	N/A	N/A	6
PACs						
PAC1	Yes	Maintenance	8 years	4 years	No	15
PAC2	Yes	Recovered sufferer	8 years	23 years	N/A	10
PAC3	Yes	Recovered sufferer	6 years	10 years	N/A	2
PAC4	Yes	N/A	N/A	N/A	N/A	1
PAC5	Yes	N/A	N/A	N/A	N/A	5
PAC6	No	N/A	N/A	N/A	N/A	8

### 2.2. Training

All coaches took part in the same training programme. We used the standard method of MI training, workshop (initial 3-day intervention with 2 × two-day booster sessions). Two trainers administered the programme: (1) a professional who has worked in the field of EDs for 25 years and is trained in MI (primary trainer – G.T.) and (2) one of the first authors of this paper (assistant trainer – P.M.). Regular supervision was given using feedback from audio-taped sessions, which were screened for overall content and adherence to the principles of MI using the Motivational Interviewing Treatment Integrity (MITI) rating system, described in the following section (Moyers, Martin, Manuel, Miller, & Ernst, 2010). Peer supervision was also used whereby coaches would rate transcripts for each other using the MITI procedure. Face-to-face supervision sessions took place both at the beginning of the programme and at regular intervals thereafter. Trainers were also immediately available by telephone to discuss any challenging situation that arose. Group email supervision was also used where a difficult scenario would be discussed confidentially and anonymously among the coaches. The team leader (J.T.), a Consultant Psychiatrist with long-standing years of experience in the field of EDs, was informed of any potentially challenging situations.

**Figure 1.**
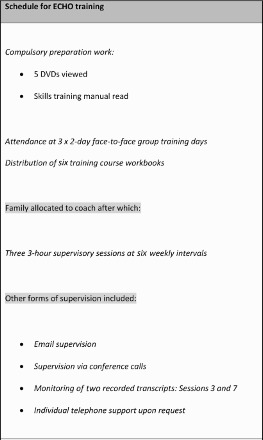
Training protocol.

The coaches were reimbursed for their travelling expenses when attending training and paid a small fee per telephone session to cover costs involved. Training days, however, were on a voluntary basis in terms of time required. Trainers suggested coaches aimed for a 40-minute telephone session. Coaches were provided with recording equipment for supervision and monitoring purposes. Coaches were initially allocated one family and once comfortable with the process, they could choose to work with up to a maximum of three families at any one time. Although individual telephone/email support was available upon request, coaches made greater use of peer discussion where scenarios were discussed by email, all identifiable information having been removed, and feedback given. Feedback was closely monitored by the trainers and was found to be the most cost- and-time-effective means of supervision. Figure 1 provides details of the training protocol.

### 2.3. Monitoring and fidelity

The MITI Code 3.1.1 is a validated and reliable behavioural coding system used to measure treatment integrity in MI (Moyers et al., 2010). The MITI consists of two components related to measurement of proficiency: global scores and behaviour counts. According to Miller and Rollnick, there is no predetermined adequate dose of training in MI. As a provisional guideline, they suggest two levels of target criteria when developing skill in MI: (1) basic competency and (2) proficiency (Miller & Rollnick, 2013). These are the criteria we have used in this study. Every coaching session was recorded for monitoring purposes. IDs were used to ensure confidentiality at all times. One family per coach was selected at random and two sessions (Sessions 3 and 7) from their available tape recordings were coded. Raters listened to a selected 20-minute segment randomly selected from the session. The first and last five minutes of the tape were not included to increase the potency of the rated selection (Waltz, Addis, Koerner, & Jacobson, 1993).

### 2.4. Coding

For research purposes, two Ph.D. students (R.H. and C.R.) were trained to code the session segments using the MITI. This training included studying the MITI training materials, a half-day face-to-face training and subsequent supervision, i.e. coding transcribed practice session segments and comparing them with another trained coder (P.M.) who holds a Ph.D. in the field of EDs. Supervision in the use of the MITI was given by J.T., a trained trainer. Telephone conference calls between the three raters took place after every five transcripts to ensure coding consistency and adherence to MITI requirements. Transcripts (*n* = 22) were coded on a standardised form. Each utterance was coded using the MITI and a behaviour count score calculated based on the number of times the coach gives information, uses MI adherent (MIA) behaviours (asking permission, affirming, emphasising control and support) and MI non-adherent (MINA) behaviours (advising, confronting and directing), the number of closed question (CQ) and open question (OQ) and the number of simple reflection and complex reflection (RC). The coder then provides two global ratings which are empathy (autonomy/support), ranging from 1 (low) to 5 (high) and spirit (evocation/collaboration), ranging from 1 (low) to 5 (high). Each transcript took approximately 30 minutes to code.

All transcripts were triple-coded. An average score from the three coders scoring sheets were used in calculating the summary scores as presented in the MITI. The following served as outcome measures for determining competence in MI along with calculation formulas (Moyers et al., 2010):
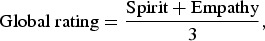


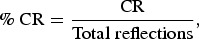


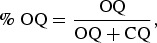


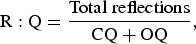


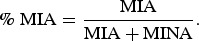



### 2.5. Data analysis

Data were analysed using SPSS 22.0. The dependent variables were the outcomes of the MITI (global rating, CRs, OQs, reflection-to-question ratio (R:O) and overall MIA) and the independent variable was coach affiliation (CCs compared to professional affiliation coaches (PACs)). *T*-test analyses were used to compare differences in treatment fidelity between those in the CC affiliation group and those in the professional coach affiliation group. The Bonferroni correction was applied to correct for the potential impact of multiple testing (5/0.05) and therefore *α* was set at 0.01. Correlational analyses were conducted using the Pearson Product Moment Coefficient to explore associations between the number of carers coached and the MITI outcome variables. Cohen's *d* (Mean 1−Mean 2/pooled standard deviation) was calculated to estimate effect sizes, with an effect size of 0.2 being defined as small, 0.5 defined as medium and 0.8 defined as large (Cohen, 1988).

## 3. Results

Over half of the sessions scored above the suggested cut-off for recommended beginning proficiency on all components of the MITI. In terms of recommended competency, half the sessions reached the cut-off for MITI global score and percentage of open questions (%OQs) and percentage MI, but did not reach the cut-off for percentage of complex reflections (%CRs) or ratio of reflections to questions. The overall MITI components for the coded sessions are displayed in Table 2.

**Table 2.** MITI scores.

MITI component	Coach affiliation	MITI recommended beginning proficiency (%)	MITI recommended competency (%)
Globals	CC	40	30
	PAC	83	67
	Total	64	50
%CR	CC	60	30
	PAC	67	17
	Total	64	23
%OQ	CC	60	20
	PAC	100	75
	Total	82	50
R:O	CC	80	20
	PAC	92	42
	Total	86	32
%MIA	CC	60	30
	PAC	83	67
	Total	73	50

### 3.1. Coach affiliation

There was a significant difference between coach affiliation on global scores, *t*(20) = 2.738, *p* = .01, effect size = 1.18, with CCs scoring significantly lower than PACs (*M* = 3.1 [1.06] and 4.18 [0.78], respectively). However, 40% (*n* = 4) of the sessions for CCs still reached the cut-off point for recommended beginning proficiency. There was also a significant difference between coach affiliation on %OQs, *t*(20) = 3.893, *p* = .001, effect size = 1.67, with CCs scoring significantly lower than their professional counterparts (*M* = 46.3 [24.26] and 81.25 [17.82], respectively). Nevertheless, 60% of the sessions for CCs still reached the cut-off point for recommended beginning proficiency. There were no significant differences for CRs, R:O or overall MIA.

For the CCs, the number of families coached was significantly correlated with their MITI global score, *r*(20) = 0.605, *p* < .05 and the percentage of MI adherent (%MIA) score, *r*(20) = 0.601, *p* < .05. The scores in both cases improved as the number of families coached increased. However, there were no such significant correlations in the PAC group. Moreover, given the small sample size, power is limited to detect effects of any size.

## 4. Discussion

The aim of this study was to examine the effectiveness of a training and supervision procedure in terms of coaching quality and fidelity to MI and to examine whether coaches with varied affiliations to EDs (those with lived and/or professional expertise in EDs) can be trained to an acceptable level of MI proficiency. Fifty per cent (*n* = 11) of the sessions had a MITI global score for spirit above the suggested cut-off for recommended competency and 64% (*n* = 14) achieved the recommended proficiency. As might be expected, individuals with prior psychological training had higher levels of treatment fidelity. The results also suggest that experiential training is associated with higher scores in the lay CCs, i.e. there is a learning curve and skills and integrity appear to improve as the number of families coached increases.

The importance of the quality of MI in the size of the effect of an intervention has been found in several reviews. Miller and Rollnick (2009) claim that although initial training can provide a certain headstart, real skill and comfort grow through disciplined practice with feedback and coaching from a knowledgeable guide and that it typically requires practice with feedback and coaching over time. Learners also have different levels of initial skilfulness and vary in how quickly they develop proficiency in MI (Miller & Rollnick, 2013). These conclusions partly support the results of this study in that there is a trend towards higher treatment integrity as the number of families coached increases for CCs, although this trend was not apparent with the PACs. According to Miller and Rollnick, every clinician is likely to vary on these indices from one session to the next and so, even the most proficient clinicians would not be expected to hit every benchmark on every interview. Recommended indices, for example, are intended to be used as a general measure and improvements implemented upon further research and experience (Miller & Rollnick, 2013). Additional research is necessary to further explore and determine the adequate dosage of training and supervision required for effective implementation (Zandberg & Wilson, 2013). This would be useful for both CCs and those with a professional background in psychology.

To date, training individuals to work with MI has been conducted primarily with professionals. Few studies in the literature base have reported the use of lay or peer coaches (Crane-Okada, Freeman, Ross, Kiger, & Giuliano, 2010) in health-related guidance. The current results, however, are consistent with one study we found that used MI to promote peer-to-peer support for cancer survivors (Allicock et al., 2012). Allicock et al. demonstrated that training “guides” to use MI results in their proficiency as assessed by the MITI scale and other process evaluation instruments. The authors concluded that MI can be adapted to train laypeople to provide support for groups such as cancer survivors.

### 4.1. Clinical implications and further research

The results of the present study illustrate the complexity of the skills involved in administering MI and the art in their application, which may take experience. Such skills and judgement are not easily obtained from a manual or short training programmes. In addition to technical competence, other coach qualities are of importance. Future studies are needed to establish whether coaches with higher treatment integrity produce better outcomes. In addition, it would be useful to have feedback from families receiving the intervention as to whether the background and affiliation of the coach are of importance to the carers, e.g. whether there is a preference for coaches with the lived experience of having supported a loved one with an ED or coaches with a professional affiliation to the field of EDs. Nevertheless, initial findings are promising in that they indicate that this procedure can be disseminated with limited resources and that CCs can be trained to a basic level of competency. Further exploration of a more effective means of training, monitoring and supervision will be required to maximise the quality of the intervention.

### 4.2. Limitations

This was an exploratory study to examine whether individuals from various backgrounds could be trained to deliver MI. There were several limitations to the study, most notably, the small sample size, limiting the study's power to detect effects of any size. Further research would benefit from a larger, more heterogeneous sample, particularly in terms of gender. Another limitation was that coaches were aware that Sessions 3 and 7 were to be monitored and so there may be greater effort to be more MIA during these two sessions. Consequently, for future research purposes, sessions to be monitored should be chosen randomly.

### 4.3. Conclusion

This study has provided preliminary evidence that the skills required for delivering ECHO can be disseminated to coaches with a lived experience of an ED with little or minimal levels of clinical training. Hence, the peer-support model is feasible. Although further research is needed to ascertain optimal levels of supervision and training, this type of dissemination can be a useful tool that will improve the cost effectiveness of inpatient treatment by enhancing the well-being of both carers and their loved ones. Carer support is an essential requirement in treatment programmes and the need to research cost-effective interventions continues to grow.
